# Exploration heuristics decrease during youth

**DOI:** 10.3758/s13415-022-01009-9

**Published:** 2022-05-19

**Authors:** Magda Dubois, Aislinn Bowler, Madeleine E. Moses-Payne, Johanna Habicht, Rani Moran, Nikolaus Steinbeis, Tobias U. Hauser

**Affiliations:** 1 Max Planck UCL Centre for Computational Psychiatry and Ageing Research, WC1B 5EH, London, UK; 2 Wellcome Centre for Human Neuroimaging, University College London, WC1N 3BG, London, UK; 3 Centre for Brain and Cognitive Development, Birkbeck, University of London, WC1E 7HX, London, UK; 4 UCL Institute of Cognitive Neuroscience, WC1N 3AZ, London, UK; 5 Division of Psychology and Language Sciences, University College London, WC1H 0AP, London, UK

**Keywords:** Decision-making, Exploration, Adolescence, Impulsivity

## Abstract

Deciding between exploring new avenues and exploiting known choices is central to learning, and this exploration-exploitation trade-off changes during development. Exploration is not a unitary concept, and humans deploy multiple distinct mechanisms, but little is known about their specific emergence during development. Using a previously validated task in adults, changes in exploration mechanisms were investigated between childhood (8-9 y/o, N = 26; 16 females), early (12-13 y/o, N = 38; 21 females), and late adolescence (16-17 y/o, N = 33; 19 females) in ethnically and socially diverse schools from disadvantaged areas. We find an increased usage of a computationally light exploration heuristic in younger groups, effectively accommodating their limited neurocognitive resources. Moreover, this heuristic was associated with self-reported, attention-deficit/hyperactivity disorder symptoms in this population-based sample. This study enriches our mechanistic understanding about how exploration strategies mature during development.

**Supplementary Information:**

The online version contains supplementary material available at 10.3758/s13415-022-01009-9.

## Introduction

Children are known to be very good learners despite their limited knowledge and cognitive capacity (Gopnik, 2020; Gopnik et al., 2015; Kidd & Hayden, 2015). Solving the paradox of how they achieve such rapid learning is the holy grail of artificial intelligence (Turing, 1950) and could help to identify developmental disorders that suffer from learning impairments (e.g., attention-deficit/hyperactivity disorder (ADHD), dyslexia, or dyscalculia) (Kaufmann, 2012; Luman et al., 2010; Snowling, 2014).

It is believed that increased “exploratory” behaviour children is key to this rapid acquisition of skills and knowledge (Gopnik, 2020). Exploration is traditionally operationalised as choices that forgo reward in order to gain information, which enables one to make better informed (and possibly more rewarding) decisions in the future. This often is contrasted with “exploitation,” which refers to choosing the option with the currently highest value. Arbitrating between those two options, commonly termed the exploration-exploitation dilemma, is central to efficient learning (Kidd & Hayden, 2015; Sutton & Barto, 1998).

Solving the exploration-exploitation dilemma is not trivial. Studies have demonstrated that humans rely on different strategies to decide when to explore (Dubois et al., 2021; Gershman, 2018; Wilson et al., 2014). Imagine that you are in an ice cream shop. There are plenty of different ice-cream flavours, but you can only pick one. How do you choose? Essentially your choice will depend on the strategy that you use. If you decide to exploit, you will go for the flavour that you have had many times in the past and are sure to enjoy, e.g., chocolate. If you decide to direct your choice towards information gain (i.e., directed exploration), you will choose the option associated to the highest sum of expected reward and information gain (i.e., how much you like it, but also how unsure you are about that choice), e.g., toblerone chocolate flavour over the classic chocolate one. This is usually modelled by using the upper confidence bound (UCB) algorithm (Auer, 2003; Gershman, 2018; Schulz & Gershman, 2019). A simpler form of this strategy is to simply choose a flavour that is entirely novel (i.e., novelty exploration) (Dubois et al., 2021; Stojic et al., 2020), e.g., a hibiscus ice cream. Alternatively, you could inject some randomness in the decision process and could choose an option with a probability that scales with its expected value (i.e., softmax decision function, a value-based random exploration strategy): e.g., have a high probability of choosing chocolate and a smaller probability of choosing Toblerone. The problem with this strategy is that it does not assign any probability to options for which there is not expected value, e.g., hibiscus. To solve this, it is usually combined with UCB (so that the novel option has an expected value proportional to its novelty; Gershman, 2018) or replaced by a more sophisticated version of random exploration—Thompson sampling algorithm (Thompson, 1933)—where the probability of choosing an option scales with both the expected value (how much you like it) and the uncertainty (Gershman, 2018), effectively an uncertainty-dependent value-based random exploration. A very simple way to explore would be to simply choose with “closed-eyes,” effectively assigning an equal probability to explore any option, irrespective of their expected value, e.g., equal probability of choosing hibiscus, Toblerone, or even a disgusting spinach ice cream. This can be captured using the ϵ-greedy algorithm (Sutton & Barto, 1998); we refer to it as value-free random exploration (Dubois et al., 2021).

Those exploration strategies differ in sophistication and computational demand and can engage more or less reflective process (Otto et al., 2014). At the end of this range the so-called “complex exploration strategies” require more computational resources. For example, Thompson sampling or UCB require to keep track of the expected values and uncertainties of all different options, which makes it challenging to keep track of many options at the same time. In contrast, at the other end of this range, “exploration heuristics” can be less optimal but have the advantage of requiring little cognitive resources. For example, value-free random exploration ignores all prior knowledge, and does thus not require any information be held in memory. Interestingly, alternative formulations of reducing complexity, such as policy compression (Gershman, 2020) suggest that perseveration (i.e., choosing the same option repeatedly) rather than a uniform policy (as proposed here) would reduce complexity. Another example towards the end of this range, is the novelty exploration strategy, which is heavily biased towards novelty, primarily engaging only with novel option, and which has recently been shown to be present over and above the complex exploration strategies (Dubois et al., 2021; Stojic et al., 2020). Other strategies, such as the softmax temperature, are often used in conjunction with other strategies (Gershman, 2018; Meder et al., 2021) and require to keep track of expected means but not uncertainties, probably reflect an intermediary complexity. In addition, other exploration strategies have been identified, such as win-stay-lose-shift (Wu et al., 2018) and its related win-stay-lose-sample (Bonawitz et al., 2014), count-based exploration strategies (Bellemare et al., 2016; Dezza et al., 2019) or a “local” information-seeking exploration strategy, i.e., repeated sampling of the same option for hypothesis testing (Alméras et al., 2022).

These heuristics seem to be of particular use in contexts when less cognitive resources are available: for example, in the case of an increased working memory load (Dezza et al., 2019) or under time pressure (Wu et al., 2021). This is particularly pertinent in children and adolescents given their reduced cognitive and neural capacity (Gopnik et al., 2015; Thompson-Schill et al., 2009), as reflected in limited executive functions. Indeed, although executive functions already emerge during the first years of life, they significantly expand throughout childhood and adolescence. This is for example the case for working memory, which is refined throughout adolescence and early adulthood, especially for tasks which require keeping track and manipulating multiple items (Best & Miller, 2010). Similarly, cognitive control for decision-making is known to improve during adolescence (Steinbeis & Crone, 2016). All those improvements are thought to be at least partially due to a delayed maturation of brain areas serving higher cognitive functions, such as the prefrontal cortex (PFC) (Casey et al., 2005; Hartley & Somerville, 2015; Steinbeis & Crone, 2016; Ziegler et al., 2019).

Research in humans has shown that adults supplement complex exploration strategies (e.g., UCB or Thompson sampling) with non-demanding exploration heuristics. One of those consists in inducing stochasticity during value comparison, e.g., a softmax temperature parameter (Daw et al., 2006; Schulz et al., 2018; Zajkowski et al., 2017), which is usually combined with UCB (Daw et al., 2006; Wilson et al., 2014). We have recently shown that adults supplement complex exploration strategies with two nondemanding exploration heuristics (Dubois et al., 2021): value-free random exploration and novelty exploration. Value-free random exploration (algorithmically captured by ϵ-greedy; Sutton & Barto, 1998) is the cheapest way to explore whereby one ignores all prior information and chooses all options with an equal probability. In effect, as opposed to the above-mentioned value-based random exploration, in this regime stochasticity is added independently of value computation. Novelty exploration is another heuristic whereby only options not encountered previously are chosen. This captures the intrinsic value of choosing something new by adding a novelty bonus (Krebs et al., 2009) to previously unseen choice options. This may be particularly useful when generalizing knowledge to more uncertain environments (Stojic et al., 2020).

Exploration is thought to be high in children and to diminish as they grow older (Blanco & Sloutsky, 2021; Gopnik, 2020; Gopnik et al., 2015; Liquin & Gopnik, 2020), which stands in stark contrast to the limited resources that developing youths have for sophisticated problem solving. A solution for this paradox could be that they use such (phylogenetically old) heuristic strategies, which may not be optimal but efficient under given constraints. Experimentally, however, evidence for this hypothesis is limited. Previous studies have found differences in computationally complex strategies, such as a change in the valuation of uncertainty in UCB exploration (Schulz et al., 2019) and a change in the strategic usage of directed exploration (i.e., a larger horizon modulation) (Somerville et al., 2017) between children and adults, but none have considered the utilisation of simpler heuristics as an add-on to complex strategies and how they develop before adulthood. We hypothesise that value-free random exploration, or in other words computationally cheap exploration without integrating prior knowledge, plays a particularly crucial role at a young age.

Understanding the developmental trajectories of exploration strategies also may be relevant for understanding developmental psychiatric disorders. Previous studies have shown that excessive exploration is a mechanism underlying attention-deficit/hyperactivity disorder (ADHD) (Addicott et al., 2020; Hauser et al., 2014; Hauser et al., 2016); however, it is unclear which specific exploration strategy is involved. The noradrenaline-modulated and computationally inexpensive value-free random exploration (Dubois et al., 2021) is a good candidate, as mental-effort is less readily invested by impulsive subjects (Patzelt et al., 2019), and because noradrenaline is a critical contributor to ADHD (Arnsten & Pliszka, 2011; Berridge & Devilbiss, 2011; Del Campo et al., 2011; Frank et al., 2007; Hauser et al., 2016).

To test our hypotheses, we developed and validated a child-friendly, apple-gathering task (Dubois et al., 2021; Dubois & Hauser, 2021), which allowed us to tease apart the contributions of complex exploration strategies and exploration heuristics. The task is an extended and modified three-option variant of the well validated horizon task (Wilson et al., 2014), which was made child-friendly by using apples of varying size (instead of numbers) and with highly engaging visuals. Importantly, we developed the tasks to capture and dissociate these different complex and simple exploration strategies (Dubois et al., 2021). Using behavioural markers and computational modelling, we found that younger age groups displayed an increased deployment of value-free random exploration. In addition, we find that subjects scoring higher on ADHD symptoms rely more on value-free random exploration.

## Methods

### Subjects

We recruited 108 subjects from schools in Greater London. Eleven subjects were excluded from the analysis: 10 due to incomplete data collection (technical issues) and 1 due to a preexisting medical condition. The final sample consisted of 26 children (16 females; age: mean [M] = 9.32 years, standard deviation [SD] = 0.27, range = 8.89-9.71), 38 early adolescents (21 females; age: M = 13.13 years, SD = 0.30, range = 12.69-13.64), and 33 late adolescents (19 females; age: M = 17.18 years, SD = 0.29, range = 16.71–17.45).

The sample was collected deliberately from schools in disadvantaged areas to oppose the common recruiting bias of participants from high socioeconomic status (Fakkel et al., 2020) and to likely increase the variability of ADHD symptom scores. We determined the sample size assuming effect sizes (medium to large) comparable to our previous study using the same task (Dubois et al., 2021) and to previous developmental studies, which have found meaningful developmental differences across age groups (Bowler et al., 2021; Decker et al., 2015; Eppinger et al., 2013; Rodriguez Buritica et al., 2019; Tymula et al., 2012; Unger et al., 2016; Weil et al., 2013). Our power simulations revealed that a sample size of around 30 subjects per group is enough to reach a statistical power or 80% (Fig. S2 for details about power simulations). Age groups did not differ in gender or intellectual abilities (Table S1 in the Supplemental Material). Each subject was given a gift voucher of £7 but did not get any additional monetary incentives for task performance.

As a measure of ADHD symptoms, we used the Self-Report Conners 3 ADHD Index (Conners 3AI-SR, adjusted for age and gender; Conners, 2008). The Conners 3AI-SR is an index that contains the 10 items from the full-length Conners 3 questionnaire, which best differentiate youths with ADHD from youths in the general population (Conners, 2008). All age groups had similar ADHD scores (Table S1 in Supplementary Materials). All subjects provided written, informed consent, and everyone younger than age 16 years provided written permission from a parent or legal guardian. The UCL research ethics committee approved the study.

### Task

**Fig. 1:**
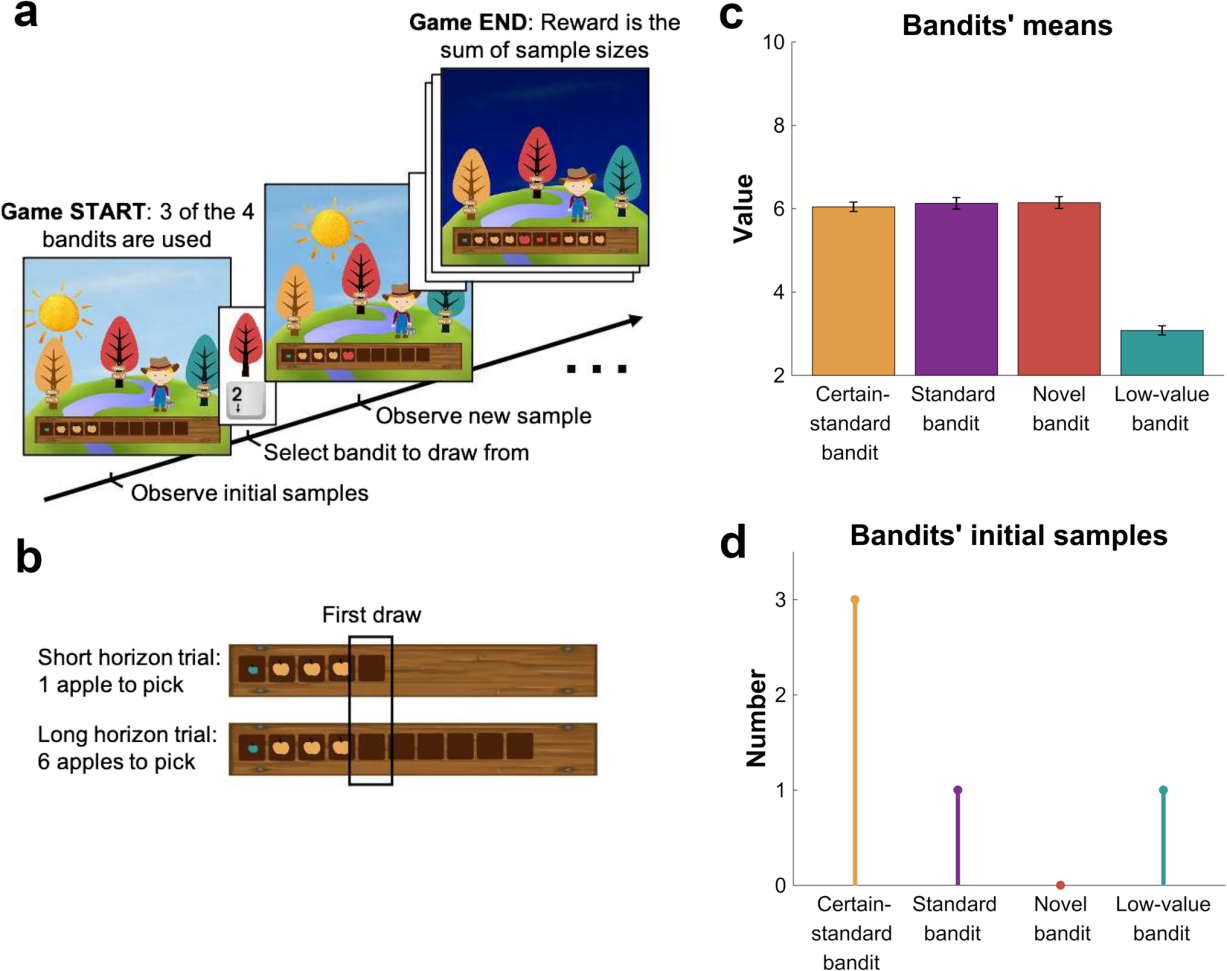
Exploration task. In the Maggie’s farm task, subjects had to choose from three bandits (depicted as trees) to maximise their overall reward. The rewards (apple size) of each bandit followed a normal distribution with a fixed sampling variance. (**a**) At the beginning of each trial, subjects are provided with some initial samples (number varied depending on the bandits present on that trial) on the wooden crate at the bottom of the screen and subjects had to select which bandit they want to sample from next. (**b**) Depending on the condition, they can either make one draw (short horizon) or six draws (long horizon). The empty spaces on the wooden crate (and the position of the sun) indicate how many draws they have left. Bandits could be of four generative groups characterized by different (**c**) mean reward and (**d**) number of initial samples (Methods for details)

To capture different forms of exploration, we used the previously validated Maggie’s Farm task (Dubois et al., 2021) (Fig. 1), an extended and modified three-option variant of a previously developed “Horizon” task (Wilson et al., 2014). In this task, subjects had to choose to draw a sample from (i.e., pick an apple) between different bandits (depicted as trees; Fig. 1a) to maximise a sum of reward (represented by the apples’ size; Fig. S1 in Supplementary Materials). To help them with their decision, at the beginning of each trial, subjects had some information about how good each bandit was in the form of “initial samples” (i.e., apples that have been picked before). Bandits carried either a lot, some, or no prior information (i.e., 3, 1 or 0 initials samples; Fig. 1d) and had either a standard or a low reward mean (Fig. 1c). In effect, there were four different types of bandits: the certain-standard bandit (standard mean, 3 initial samples), the standard bandit (standard mean, 1 initial sample), the novel bandit (standard mean, 0 initial samples), and the low-value bandit (low mean, 1 initial sample). On each trial, three of those four bandit types were used. In the analysis, the bandit with the highest mean reward of prior samples (either 1 or 3) is referred to as the “high-value bandit.”

This task allows to distinguish between complex exploration strategies and exploration heuristics, namely, value-free random exploration and novelty exploration (see Methods for details). Manipulating the number of prior samples and reward allows to capture complex exploration strategies as they take expected values and uncertainty into account. Value-free random exploration is a computationally very light heuristic that does not take any prior knowledge into account. In effect, it chooses randomly between options, which can lead to choosing any option, even those known to be bad (e.g., associated to a low reward prior sample), such as the low-value bandit. Because the low-value bandit will primarily by chosen under this regime, it allows to quantify the contribution of value-free random exploration. Similarly, the novel bandit allows to capture novelty exploration, a heuristic that targets entirely novel options.

To promote and assess exploration, we manipulated the number of choices per trial (i.e., decision horizon; Fig. 1b). Subjects could perform either one draw, encouraging exploitation (short horizon condition), or six draws, encouraging more substantial explorative behaviour (long horizon condition), because in the latter condition, the newly gained information could subsequently be exploited. If not stated otherwise, we compare the short horizon’s single draw to the long horizon’s first draw in alignment with previous studies by using the same manipulation (Dubois et al., 2021; Wilson et al., 2014). Subjects performed a training session to make sure they understood the instructions and the general concept before playing a total of 96 trials (48 in each horizon condition) during the task.

### Statistical Analyses

We compared behavioural measures and model parameters using repeated-measures ANOVAs with the age group as between-subject factor (children, early adolescents, late adolescents), the decision horizon as within-subject factor horizon (long, short horizon). Bonferroni correction was applied for multiple comparisons with N = 3 for exploration strategies captured by model parameters and N = 3 for behavioural measures (i.e., bandits). We additionally performed the analyses when correcting for IQ (by adding IQ scores as covariate in the ANOVAs). We report effect sizes using partial eta squared (η^2^) for ANOVAs and Cohen’s d (d) for *t*-tests. Post hoc tests were conducted using paired and independent sample *t*-test applying Bonferroni correction for multiple comparisons. To assess correlations between ADHD symptoms and exploration strategies, the exploration strategy parameters were averaged across horizon. We performed both bivariate and partial correlations (correcting for age and IQ) using Pearson correlation.

### Computational Modelling

We compared a set of generative models which assumed different exploration strategies accounting for subjects’ behaviour. Three core models were examined: UCB, Thompson sampling, and a hybrid of these two. The UCB model captures directed and value-based random exploration, whereas the Thompson model captures an uncertainty-driven, value-based exploration. In both UCB and Thompson sampling, the stochasticity is added during value comparison (respectively in the logistic sigmoid function and in the probit sigmoid function), but in the former the stochasticity is fixed (i.e., the softmax decision temperature free parameter), while in the latter the stochasticity scales with the total uncertainty (see Gershman, 2018 for details). The hybrid model combines all of the above. We computed three extensions of each model by either adding value-free random exploration, novelty exploration or both heuristics, leading to a total of 12 models. They key motivation for this model comparison was to assess whether the exploration heuristics (novelty, value-free random exploration) exist in addition to a complex (UCB, Thompson, or both) model (Supplementary Material for details about the models).

## Results

### Subjects Increase Exploration when Information can Subsequently be Exploited

In this exploration task we manipulated the number of apples to be picked on each trial to encourage exploration (Dubois et al., 2021). In the long horizon, six different apples could be picked in sequence, which promotes initial exploration because gaining new information could improve later choices.

**Fig. 2:**
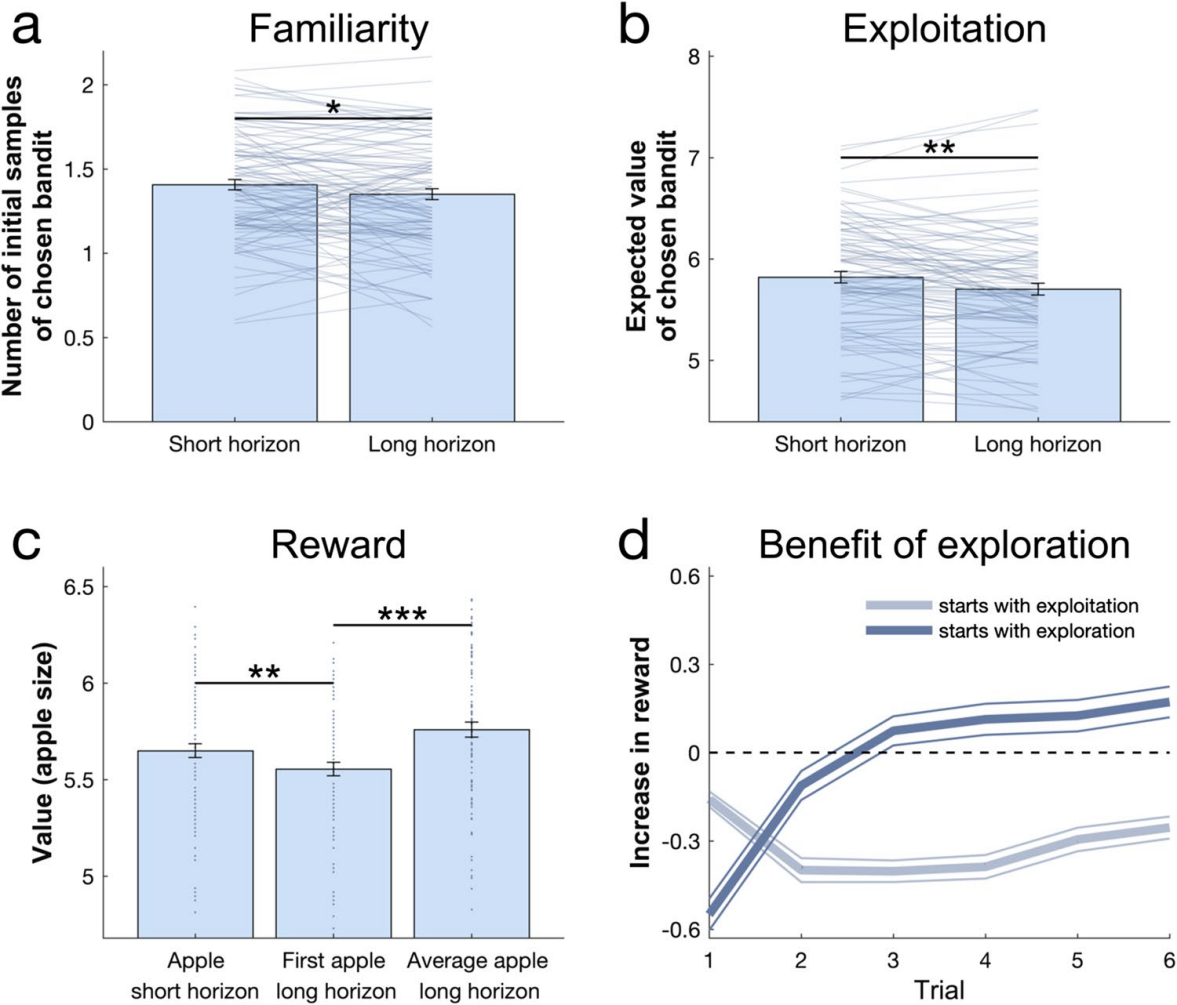
Benefits of exploration. (**a**) Subjects (collapsed across all age groups) chose less familiar (i.e., more informative) bandits on their first choice in the long compared to the short horizon. (**b**) Subjects chose bandits with a lower expected value (i.e., they exploited less) in the long horizon compared to the short horizon. (**c**) This behaviour led to a lower reward in the long horizon than in the short horizon on their first draw, indicating that subjects sacrificed larger initial outcomes for the benefit of more information. This additional information helped making better decisions in the long run, leading to higher earnings over all draws in the long horizon (right bar plot). Similarly, (**d**) in the long horizon, starting off by exploring (dark blue) versus exploiting (choosing the bandit with the highest expected value; light blue), led to an initial decrease in reward (negative increase in reward; difference between obtained reward and highest reward of initial samples), but eventually increased it. This means that the information that was gained through exploration led to higher long-term outcomes. *p < 0.05; **p < 0.01; ***p < 0.001. Data are mean ± SEM and each dot/line represent a subject

To assess whether a longer decision horizon promoted exploration in our task, we compared which bandit subjects chose in their first draw in the short and in the long horizon condition. For each trial we computed the familiarity (the mean number of initial samples shown) and the expected value (the mean value of initial samples shown) of each bandit. In the long horizon condition, subjects preferred less familiar bandits (horizon main effect: F(1, 94) = 5.824, *p* = 0.018, η^2^ = 0.058; age main effect: F(2, 94) = 0.306, *p* = 0.737, η^2^ = 0.006; age-by-horizon interaction: F(2, 94) = 0.836, *p* = 0.436, η^2^ = 0.017; Fig. 2a), even at the expense of it having a lower expected value (horizon main effect: F(1, 94) = 11.857, *p* = 0.001, η^2^ = 0.112; age main effect: F(2, 94) = 2.389, *p* = 0.097, η^2^ = 0.048; age-by-horizon interaction: F(2, 94) = 0.031, *p* = 0.969, η^2^ = 0.001; Fig. 2b). This is mainly driven by the fact that subjects selected the high-value bandit (i.e., the bandit with the highest expected reward based on the initial samples) less often in the long horizon (horizon main effect: F(1, 94) = 24.315, *p* < 0.001, η^2^ = 0.206; age main effect: F(2, 94) = 1.627, p_cor_ = 0.808, p_unc_ = 0.202, η^2^ = 0.033; age-by-horizon interaction: F(2, 94) = 2.413, *p* = 0.095, η^2^ = 0.049; Fig. 4a; when adding IQ as a covariate: horizon main effect: F(1,94) = 24.017, *p* < 0.001, η^2^ = 0.204; age main effect: F(1,94) = 2.183, p_cor_ = 0.429, p_unc_ = 0.143, η^2^ = 0.023; age-by-horizon interaction: F(1,94) = 2.462, *p* = 0.12, η^2^ = 0.026), demonstrating a reduction in exploitation when information can subsequently be used. This behaviour resulted in a lower initial reward (on the 1^st^ sample) in the long compared with the short horizon (1^st^ sample: horizon main effect: F(1, 94) = 13.874, *p* < 0.001, η^2^ = 0.129; age main effect: F(2, 94) = 1.752, *p* = 0.179, η^2^ = 0.036; age-by-horizon interaction: F(2, 94) = 1.167, *p* = 0.316, η^2^ = 0.024; Fig. 2c).

To evaluate whether subjects used the additional information in the long horizon condition beneficially, we compared the average reward (across six draws) obtained in the long compared to short horizon (one draw). The average reward was higher in the long horizon (horizon main effect: F(1, 94) = 17.757, *p* < 0.001, η^2^ = 0.159; age main effect: F(2, 94) = 2.945, *p* = 0.057, η^2^ = 0.059; age-by-horizon interaction: F(2, 94) = 0.555, *p* = 0.576, η^2^ = 0.012; Fig. 2c), indicating subjects tended to choose less optimal bandits at first but subsequently made use of the harvested information to guide a choice of better bandits in the long run. This also was the case when we looked at the long horizon exclusively and compared the increase in reward (difference between the obtained reward and the highest shown reward) between when subjects started with an exploitative choice (chose the bandit with the highest expected value) versus an exploratory one. Exploration decreased their reward at first (long horizon 1^st^ choice: exploration main effect: F(1, 94) = 39.386, *p* < 0.001, η^2^ = 0.295; age main effect: F(2, 94) = 0.443, *p* = 0.643, η^2^ = 0.009; age-by-exploration interaction: F(2, 94) = 0.433, *p* = 0.650, η^2^ = 0.009; Fig. 2d), but eventually increased it (long horizon 6^th^ choice: exploration main effect: F(1, 94) = 63.830, *p* < 0.001, η^2^ = 0.404; age main effect: F(2, 94) = 1.820, *p* = 0.168, η^2^ = 0.037; age-by-exploration interaction: F(2, 94) = 0.753, *p* = 0.474, η^2^ = 0.016; Fig. 2d), indicating that they were able to take advantage of the information gained through exploration.

### Subjects explore using computationally expensive strategies and simple heuristics

**Fig. 3:**
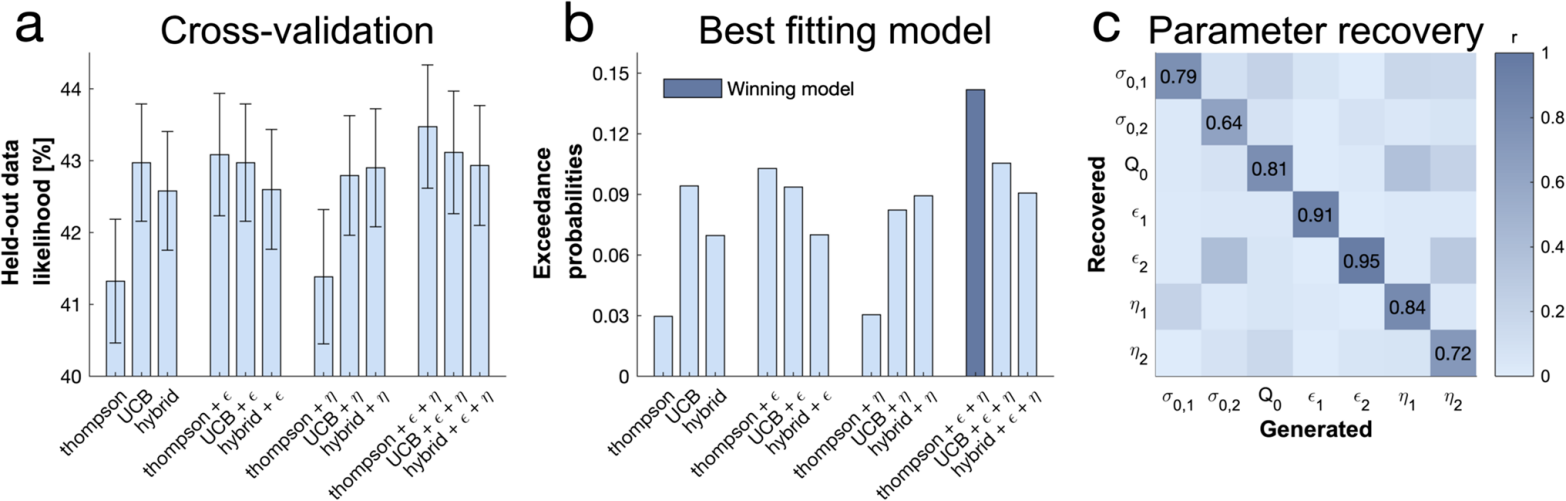
Subjects use a mixture of exploration strategies. A sixfold cross-validation of the likelihood of held-out data was used for model selection. The labels on the x-axis capture the contributions of different models: uncertainty-dependent value-based random exploration (Thompson, i.e., Thompson sampling algorithm), directed exploration and value-based random exploration (UCB, i.e., UCB algorithm combined with a softmax decision function), a mixture of those three (hybrid, i.e., combined UCB and thompson), value-free random exploration (ϵ, i.e., ϵ-greedy parameter) and novelty exploration (η, i.e., novelty bonus parameter). (a) The average held-out data likelihood with standard error of the mean (SEM) bars. (b) The exceedance probabilities when using Bayesian model selection predicted the winning model: the Thompson model with both the ϵ-greedy parameter and the novelty bonus η. (c) Model simulation with 4^7^ simulations predicted good recoverability of model parameters; σ_0_ is the prior variance and Q_0_ is the prior mean (cf. Supplementary Material for details about the models); 1 stands for short horizon-, and 2 for long horizon-specific parameters

To determine which exploration strategies subjects use, we compared 12 models (cf. Supplementary Materials) using K-fold cross-validation. Essentially, the data of each subject is partitioned into K folds (i.e., subsamples). Each model is fitted to K-1 folds and validated on the remaining fold (i.e., held-out data). This process is repeated K times so that each of the K folds is used as a validation set once. The model with the highest average likelihood of held-out data is then selected as the winning model. During model selection, we compared a UCB model (directed exploration and value-based random exploration), a Thompson model (uncertainty-driven value-based exploration), a hybrid of both and a combination of those with an ϵ-greedy (value-free random exploration) and/or a novelty bonus (novelty exploration). These models made different predictions about how an agent explores and makes the first draw in each trial. Using Thompson sampling (Gershman, 2018; Thompson, 1933; captured by the Thompson model), she takes both expected value and uncertainty into account, with higher uncertainty leading to more exploration (uncertainty-driven value-based exploration). Using the UCB algorithm (Auer, 2003; Gershman, 2018; part of the UCB model), she also takes both into account but chooses the bandit with the highest (additive) combination of expected information gain and reward value (directed exploration). This computation is then passed through a softmax decision function inducing so-called value-guided random exploration. The novelty bonus is a simplified version of the information bonus in UCB, which only applies to entirely novel options (novelty exploration). Using ϵ-greedy, a bandit is chosen entirely randomly, irrespective of expected values and uncertainties (i.e., value-free random exploration). Similarly to previous studies in adults (Dubois et al., 2021; Dubois & Hauser, 2021), we found that subjects used a mixture of computationally demanding strategies (i.e., Thompson sampling or UCB) and two heuristic exploration strategies (i.e., ϵ-greedy and the novelty bonus), as captured by the model comparison (paired-samples *t*-test: 1^st^ model: Thompson+*ϵ*+*η* vs. 2^nd^ model: UCB+*ϵ*+*η*: t(96) = 1.804, *p* = 0.074, d = 0.183; 1^st^ model: Thompson+*ϵ*+*η* vs 3^rd^ model: Thompson+*ϵ*: t(96) = 2.52, *p* = 0.013, d = 0.256; Thompson+*ϵ*+*η* vs Thompson: t(96) = 6.687, *p* < 0.01, d = 0.679; Fig. 3a). The winning model was given by Bayesian Model Selection (Fig. 3b; cf. Supplementary Materials for more details). Simulations revealed that the winning model’s parameter estimates could be accurately recovered (Fig. 3c).

### Value-Free Random Exploration Decreases in Late Adolescents

**Fig. 4:**
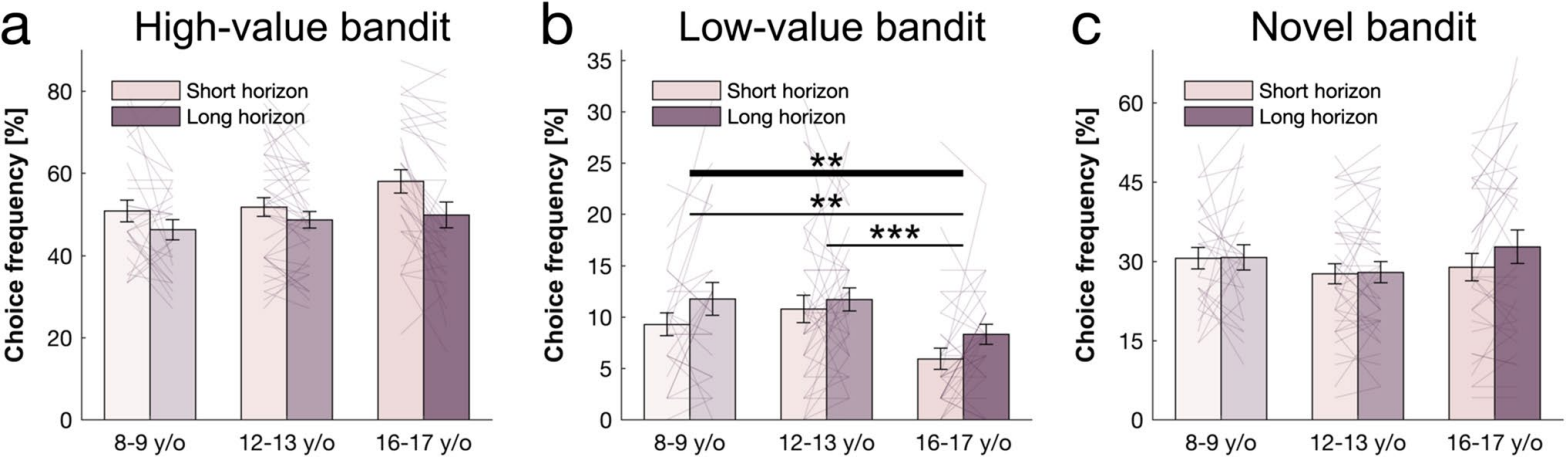
Behavioural age effects. Choice patterns in the first draw for each horizon and age group (children: ages 8 and 9 years, early adolescents: ages 12 and 13 years, late adolescents: ages 16 and 17 years). (**a**) Early and late adolescents, but not children, sampled from the high-value bandit (i.e., bandit with the highest average reward of initial samples) more in the short horizon compared to the long horizon, showing that the horizon manipulation altered exploration behaviour. (**b**) Late adolescents sampled less from the low-valued bandit compared to the children and early adolescents indicating that value-free random exploration is reduced midway through adolescence. (**c**) Age groups did not differ in the amount of novelty exploration as measured by the choice frequency of the novel bandit, although it seems that its modulation by the horizon emerges in the old adolescent group. Horizontal bars represent rm-ANOVA (thick) and pairwise comparisons (thin). *p < 0.05; **p < 0.01; ***p < 0.001. Data are mean ± 1 SEM and each line represent one subject

**Fig. 5:**
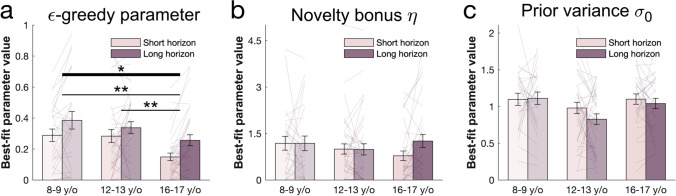
Age effects on model parameters. The winning model’s parameters were fitted to each subject’s first draw. (**a**) Late adolescents had lower values of ϵ (value-free random exploration) overall compared to children and early adolescents, indicating that value-free random exploration decreases during adolescence. Children and late adolescents had higher values of ϵ in the long compared to the short horizon. (**b**) Subjects from all groups assigned a similar value to novelty, captured by the novelty bonus η. It was higher (more novelty exploration) in the long compared with the short horizon for late adolescents only indicating a goal-directed novelty exploration. (**c**) Subjects from all groups were similarly uncertain (prior variance σ_0_ value) about a bandit’s prior mean indicating a similar uncertainty-driven value-based exploration. ^†^*p* < 0.10; *p < 0.05; **p < 0.01; ***p < 0.001. Data are mean ± SEM and each line represent one subject

Value-free random exploration (captured by ϵ-greedy) predicts that ϵ% of the time each option will have equal probability of being chosen. Under this regime, in contrast to other exploration strategies, bandits with a known low value are more likely to be chosen. To assess the deployment of this exploration form across horizons, we investigated the behavioural signature—the frequency of selecting the low-value bandit—and found that it was higher in the long compared with the short horizon condition (horizon main effect: F(1, 94) = 8.837, *p* = 0.004, η^2^ = 0.086; Fig. 4b). This also was captured more formally by analysing the fitted ϵ parameter, which was larger in the long compared to the short horizon (horizon main effect: F(1, 94) = 20.63, *p* < 0.001, η^2^ = 0.180; Fig. 5a). These results indicate that subjects made use of value-free random exploration in a goal-directed way, deploying it more when it was beneficial.

Next, we investigated our hypothesis that the age groups differed in their use of value-free random exploration usage. We thus looked at the two measures of value-free random exploration: the frequency of selecting the low-value bandit and, more formally, the ϵ-greedy parameter. We found that age groups differed in the frequency of selecting the low-value bandit (age main effect: F(2, 94) = 4.927, *p* = 0.009, η^2^ = 0.095; age-by-horizon interaction: F(2, 94) = 0.236, *p* = 0.790, η^2^ = 0.005; Fig. 4b). This also was the case when controlling for IQ (adding IQ as a covariate: age main effect: F(1,94) = 4.467, *p* = 0.037, η^2^ = 0.045; age-by-horizon interaction: F(1,94) = 0.019, *p* = 0.89, η^2^ < 0.001). Interestingly, we found that the effect was primarily driven by a reduction of selecting the low-value bandit in late adolescents, compared with early adolescents and children (children vs. late adolescents: t(52) = 2.842, p_cor_ = 0.015, p_unc_ = 0.005, d = 0.54; early vs. late adolescents: t(76) = 3.842, p_cor_ = 0.001, p_unc_ < 0.001, d = 0.634), whilst children and early adolescents did not differ (t(52) = −0.648, p_cor_ = 1, p_unc_ = 0.518, d = 0.115). This suggests that the reduction in the value-free random exploration heuristic usage occurs only later in adolescent development.

The same effect was observed when analysing the fitted ϵ parameter from the winning computational model (age main effect: F(2, 94) = 3.702, *p* = 0.028, η^2^ = 0.073; age-by-horizon interaction: F(2, 94) = 0.807, *p* = 0.449, η^2^ = 0.017; Fig. 5a). This also was the case when controlling for IQ (adding IQ as a covariate: F(1,94) = 5.583, *p* = 0.02, η^2^ = 0.056; age-by-horizon interaction: F(1,94) = 0.119, *p* = 0.73, η^2^ = 0.001). Again, this was driven by a reduced ϵ in the late adolescents compared to the younger groups (t(52) = 3.229, p_cor_ = 0.006, p_unc_ = 0.002, d = 0.622; early vs. late adolescents: t(76) = 2.982, p_cor_ = 0.009, p_unc_ = 0.003, d = 0.491; children vs. early adolescents: t(52) = 0.581, p_cor_ = 1, p_unc_ = 0.562, d = 0.105). Our findings thus suggest that, compared with late adolescents, children and early adolescents rely more strongly on the computationally simple value-free random exploration.

### No Observed Age Effect on Other Exploration Strategies

Next, we investigated whether the other exploration strategies also showed age differences, or whether value-free random exploration was the primary driver. When looking at the novelty heuristics (i.e., the tendency to select novel options), we did not observe any difference – neither in the frequency of selecting the novel bandit (age main effect: F(2, 94) = 0.341, p_cor_= 1, p_unc_ = 0.712, η^2^ = 0.007; horizon main effect: F(1, 94) = 1.534, *p* = 0.219, η^2^ = 0.016; age-by-horizon interaction: F(2, 94) = 1.522, *p* = 0.224, η^2^ = 0.031; adding IQ as a covariate: age main effect: F(1,94) = 0.014, p_cor_ = 1, p_unc_ = 0.905, η^2^ < 0.001; age-by-horizon interaction: F(1,94) = 2.227, *p* = 0.139, η^2^ = 0.023; Fig. 4c), nor more formally in the fitted novelty bonus η (age main effect: F(2, 94) = 0.341, p_cor_ = 1, p_unc_ = 0.712, η^2^ = 0.007; age-by-horizon interaction: F(2, 94) = 2.119, *p* = 0.126, η^2^ = 0.043; horizon main effect: F(1, 94) = 1.892, *p* = 0.172, η^2^ = 0.020; adding IQ as a covariate: age main effect: F(1,94) = 0.406, p_cor_ = 1, p_unc_ = 0.526, η^2^ = 0.004; age-by-horizon interaction: F(1,94) = 3.372, *p* = 0.069, η^2^ = 0.035; Fig. 5b).

Next, we assess whether there are age differences for the indicator of complex exploration strategies. We thus compared the model-derived prior variance (or uncertainty) σ_0_, which is used for the computation of the uncertainty about the expected value of each bandit (Dubois et al., 2021; Gershman, 2018). Essentially, σ_0_ is the uncertainty about the reward that subjects expect to get from a bandit before integrating its initial samples. We did not observe any difference prior variance σ_0_ (i.e., uncertainty; age main effect: F(2, 94)=3.241, p_cor_ = 0.132, p_unc_ = 0.044, η^2^ = 0.065; age-by-horizon interaction: F(2, 94) = 0.866, *p* = 0.424, η^2^ = 0.018; horizon main effect: F(1, 94) = 1.576, *p* = 0.212, η^2^ = 0.016; adding IQ as a covariate: age main effect: F(1,94) = 0.014, p_cor_ = 1, p_unc_ = 0.905, η^2^ < 0.001; age-by-horizon interaction: F(1,94) = 2.227, *p* = 0.139, η^2^ = 0.023; Fig. 5c). We were thus not able to reliably identify any other exploration strategy that changed over these developmental stages.

### Value-Free Random Exploration is Linked to ADHD Symptoms

Developmental effects on exploration strategies also are important to understand neurocognitive processes underlying developmental psychiatric disorders, such as ADHD, which has been suggested to be linked to excessive exploratory behaviour (Hauser et al., 2014; Hauser et al., 2016). A study has previously shown that value-free random exploration is a “cheap” exploration strategy modulated by noradrenaline (Dubois et al., 2021), a neurotransmitter known to be critically involved in the pathogenesis and treatment of ADHD (Arnsten & Pliszka, 2011; Berridge & Devilbiss, 2011; Del Campo et al., 2011; Frank et al., 2007; Hauser et al., 2016; Luman et al., 2010). Given that value-free random exploration stands out by its low computational demand, we hypothesized that ADHD symptoms in our population sample would be primarily linked to an over-reliance on this exploration heuristic.

**Fig. 6:**
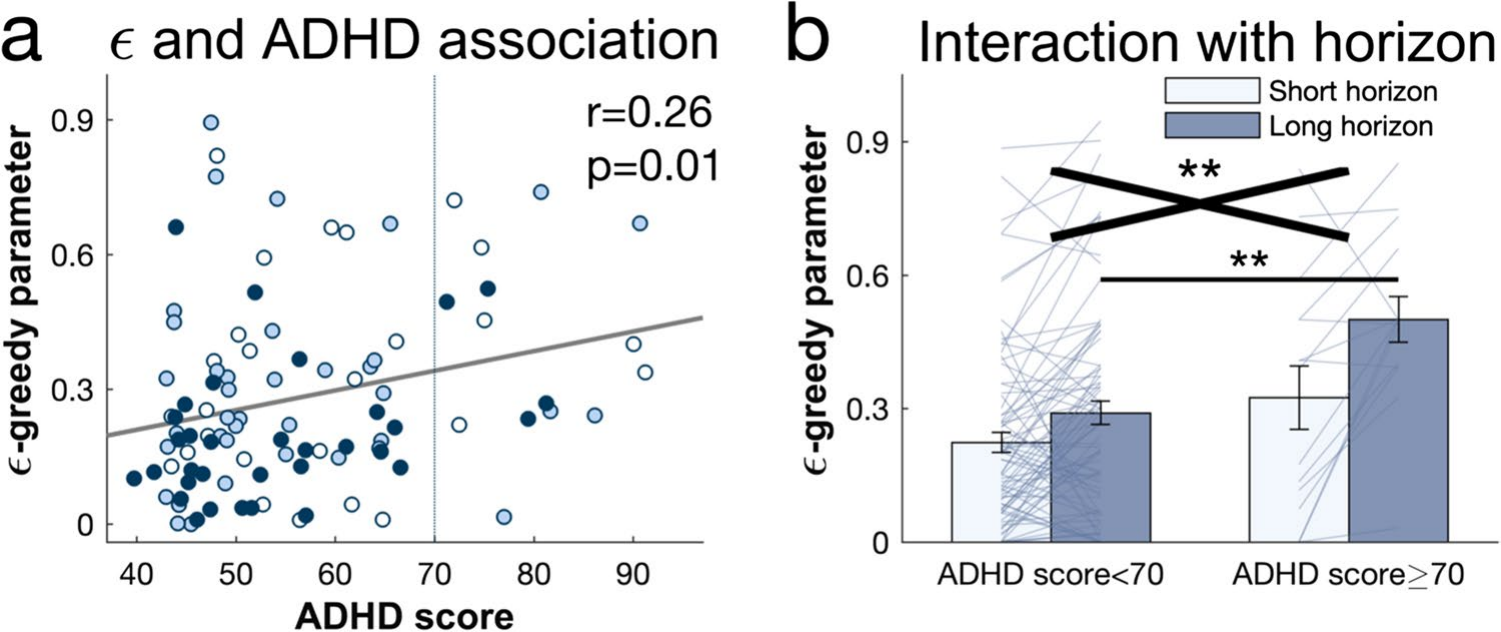
Increased value-free random exploration in ADHD. (**a**) Value-free random exploration (as captured by the model parameter ϵ) was linked to ADHD symptom scores in this population sample. A score >70 (dashed vertical line) is considered very elevated (Conners, 2008). Each dot represents one subject. White: children, light blue: early adolescents, dark blue: late adolescents. (**b**) Further analysis revealed an excessive usage of value-free random exploration specifically in the long horizon in the very elevated ADHD score group (subjects with an ADHD score ≥70). Black cross: score-by-horizon interaction. Black line: pairwise t-tests. Overall, our results suggest an overuse of value-free random exploration in ADHD. **p < 0.01; ns = *p* > 0.05. Data are mean ± 1 SEM and each line represent one subject

We thus compared whether the amount of value-free random exploration was linked to ADHD scores as measured using Conners 3 self-reports (Conners, 2008). We found that ADHD symptoms were significantly associated with value-free random exploration captured by the model parameter ϵ (bivariate Pearson correlation: r = 0.259, *p* = 0.011; Fig. 6a) and as indicated by the low-value bandit picking frequency (r = 0.259, *p* = 0.01). The effect remained significant when additionally controlling for age and IQ (partial correlation with ϵ: r = 0.212, *p* = 0.039; with low-value bandit picking: r = 0.214, *p* = 0.037).

To further investigate this and to assess potential clinical implications, we split the data comparing those subjects that scored above the clinical cutoff of T ≥ 70 (Conners, 2008) (N = 15) and those scoring below (N = 82). In line with the above correlation, we found that these subjects with a highly elevated ADHD score used the value-free random exploration more excessively (model parameter ϵ: main effect of ADHD score: F(1,95) = 7.243, *p* = 0.008, η^2^ = 0.071).

We next investigated, whether this greater reliance on value-free random exploration was used in a goal-directed manner, i.e., deploying it when exploration was useful in the long horizon. Interestingly, the high ADHD group indeed deployed this exploration heuristic primarily when it was useful, i.e., in the long horizon (score-by-horizon interaction: F(1,95) = 4.643, *p* = 0.034, η^2^ = 0.047; pairwise comparisons: long horizon: t(82) = −3.655, p_cor_ = 0.002, p_unc_ = 0.001; short horizon: t(82) = −1.355, p_cor_ = 0.386, p_unc_ = 0.193; main effect of horizon: F(1,95) = 22.926, *p* < 0.001, η^2^ = 0.194; Fig. 6b).

We thus assessed whether this increase in exploration was beneficial or detrimental for their performance. We thus compared whether the high ADHD group earned more points in the long horizon. We found that the high ADHD group performed worse than the low ADHD group, i.e., scored less points (total score in the long horizon: t(82) = 2.221, *p* = 0.040), but not in the short horizon (total score in the short horizon: t(82) = 1.569, *p* = 0.136), where they deployed the exploration heuristic to a similar degree. This suggests that the subjects scoring high on ADHD “overshot” with deploying the value-free random exploration, thus leading to a worse performance in the condition where high exploration generally leads to a better performance.

Lastly, to test the specificity this association, we tested whether other model parameters were correlated with ADHD symptoms. We did not find any association between ADHD symptoms and any of the other exploration strategies (with novelty bonus η: r = −0.113, p_cor_ = 1, p_unc_ = 0.269; with prior variance σ_0_: r = 0.01, p_cor_ = 1, p_unc_ = 0.923), suggesting that value-free random exploration is the most relevant exploration factor for ADHD symptoms.

## Discussion

Given limited neurocognitive resources (Gopnik et al., 2015; Thompson-Schill et al., 2009), how is it possible that young people are able to solve the complex and computationally demanding exploration-exploitation trade-off so successfully, and to learn at an unprecedented pace? Previous studies have shown that humans rely on different exploration strategies which vary in complexity and computational needs (Gershman, 2018; Wilson et al., 2014). Some of those strategies, exploration heuristics, bypass expensive computations (Dubois et al., 2021). In the current study, we demonstrate that children and early adolescents rely more heavily on these exploration heuristics, in particular value-free random exploration, when balancing between exploiting known and exploring less well-known options.

By assigning the same choice probability to all options, effectively suppressing the need to keep track of any expected values, value-free random exploration requires minimal computational resources. However, this computational efficiency comes as the cost of choice suboptimality, as by choosing any option, it can occasionally select options of low expected value. Despite its suboptimality, we demonstrate that this heuristic is more intensely used at an early age. Our findings suggest that the limited cognitive resources during childhood are likely to be accommodated by using computationally less demanding exploration strategies. Moreover, younger individuals may not be that negatively affected by the limitations of value-free random exploration because their limited (life) experience has not yet allowed them to build sophisticated models of the world. Specifically, the limited learning experiences means that their beliefs are more imprecise or even inaccurate. Therefore, ignoring those weak and unstable priors does not significantly penalize learning. It may even help prevent the integration of (initially) falsely rated states, essentially accounting for children’s erroneous beliefs due to lack of experience. This is in line with previous studies demonstrating the benefit of noise in decision making (Findling et al., 2019; Findling & Wyart, 2020).

Interestingly, it seems that the transition in exploration strategies is not a continuous process during development but occurs mid-way through adolescence. Children (ages 8 and 9 years) and early adolescents (ages 12 and 13 years) show similar patterns of exploration, whereas the old adolescent (ages 16 and 17 years) group differed from both younger ones. The two younger groups employ more value-free random exploration compared to the older group. Additionally, novelty exploration seems to become horizon-dependent in the older group, similarly to what is observed in adults (Dubois et al., 2021), suggesting an emergence of a goal-directed novelty exploration around this period. This is in line with the late emergence (during late adolescence) of strategic usage of information for exploration (Somerville et al., 2016). This adds to the number of cognitive abilities that improve during adolescence (Gopnik, 2020; Luna et al., 2004; Waber et al., 2007) and corresponds to brain maturation in that period (Geidd, 2004; Giorgio et al., 2010; Gogtay et al., 2004; Tamnes et al., 2010), in particular the PFC (Casey et al., 2005; Segalowitz & Davies, 2004), which is essential to integrate complex sources of information required for advanced decision-making (Hartley & Somerville, 2015). This late maturation, corresponding to an increase in complex information integration (Chrysikou et al., 2013; Gopnik et al., 2015), is thought to be responsible for the slow calibration of executive function during development (Anderson, 2002; Blakemore & Choudhury, 2006; Diamond, 2009). Because those regions are less accessible or not functioning optimally in younger individuals, they might circumvent this problem by the use of less-resource demanding strategies (i.e., heuristics) for exploration, and switch to more complex strategies as they grow older.

We found that value-free random exploration was more present in subjects with increased ADHD symptoms, irrespective of their age. ADHD is believed to be linked to an impairment in the dopaminergic and noradrenergic systems (Arnsten & Pliszka, 2011; Berridge & Devilbiss, 2011; Del Campo et al., 2011; Frank et al., 2007; Hauser et al., 2016; Luman et al., 2010) with common ADHD medication targeting dopamine (e.g., methylphenidate; Iversen, 2006) and noradrenaline functioning (e.g., atomoxetine; Levy, 2008). Our previous study showed that value-free random exploration is modulated by noradrenaline (Dubois et al., 2021), and interestingly it seems to be specifically this form of exploration which is associated with ADHD in our study. This suggests that it might be the impairment in noradrenaline which underlies the increase of value-free random exploration in ADHD. Our finding thus extends previous work (Hauser et al., 2014), where an altered exploration-related behaviour was found in adolescents with ADHD, but it was unclear which type of exploration or which type of neurotransmitter was affected. Our results thus also extend a recent study demonstrating more exploration in ADHD participants (Addicott et al., 2020). Additionally, they indicate a goal-directed excessive usage of value-free random exploration (i.e., specifically in the context where exploration is useful), suggesting that the aberrant decision-making in ADHD is not simply guided mistakes. Our results help understand ADHD symptomatology, both from a computational and evolutionary perspective, i.e., in what environment it can be adaptive (Williams & Taylor, 2006), thus providing new insights into the mechanisms of ADHD. Future studies could make use of a similar paradigm in a longitudinal setup to understand the individual trajectories of exploration and how ADHD symptoms and value-free random exploration influence each other.

Exploration is an essential part of learning (Gopnik, 2020; Kidd & Hayden, 2015; Sutton & Barto, 1998) and thus crucial for development. In this study, we show substantial changes in exploration between childhood and adolescence. Our results thus clearly expand the previous studies that either only focused on exploration in children and infants (Bonawitz et al., 2011; Bonawitz et al., 2012; Cook et al., 2011; Gweon et al., 2014; Meder et al., 2021; Pelz et al., 2015), or compared adults to minors without tracing development throughout childhood and adolescence (Schulz et al., 2019; Somerville et al., 2017). Moreover, to our knowledge we are the first to compare between multiple computationally complex and simple exploration strategies and show a specific development of value-free random exploration in the transition from childhood to adolescence. Our findings thus demonstrate that youths deploy a multitude of exploration strategies and that the reliance on these strategies dynamically changes before reaching adulthood.

Value-free random exploration is used early in development because it does not rely on heavy computation skills, which only mature later on (Gopnik et al., 2015; Thompson-Schill et al., 2009). This is in line with the fact that it relies on noradrenaline functioning (Dubois et al., 2021), as noradrenaline is expressed during early stages of development (Saboory et al., 2020), making it available for use from a very young age. Additionally, the noradrenergic system is an old and well-preserved neurotransmitter system across evolution (Bauknecht & Jékely, 2017; Kass-Simon & Pierobon, 2007), suggesting that value-free random exploration may be a phylogenetically old strategy.

Interestingly, ADHD scores were weakly correlated with model fit, meaning that subjects with higher ADHD scores had a somewhat lower (absolute) model fit. A lower model fit, however, does not mean that the model is worse per se for these subjects (especially if the relative performance compared with other models remains similar). Rather, it can be the consequence of a somewhat more stochastic overall responding. A most recent study (Moutoussis et al., 2021) indeed identified a cognitive acuity factor across multiple computational tasks, which also was accounting for difference in absolute model fit.

Importantly, whilst such a value-free random exploration strategy can be useful for exploration under certain circumstances, the question arises how specific this is and whether this does not simply capture inattention, especially with relation to ADHD. In our data, we observe that this form of exploration is condition specific, i.e. it increases in the long vs short horizon condition both in the behavioural measure (frequency of picking the low-value bandit) and the model parameter (ϵ-greedy parameter). Such a condition-specific effect is unlikely to be explained by mere inattention. This is particularly relevant for our ADHD finding, where we find the effect primarily in the long (but not in the short) horizon. Similar to previous studies using comparable tasks (Somerville et al., 2016; Wilson et al., 2014; Wu et al., 2018; Zajkowski et al., 2017), a single model was used to model both decision horizon conditions. However, future studies could attempt to fit both horizons separately to see whether the model used is context dependent. It is important to note that in this sample the two complex exploration strategies (i.e., UCB and Thompson) were difficult to distinguish. However, they were associated to similar heuristics usage, which is what we were interested in here.

Taken together, our results suggest that value-free random exploration is a simple exploration strategy that is of great benefit when cognitive resources are still limited during earlier stages of development. As we grow older and our experience expands, it is evolutionary useful to incorporate our knowledge in decision making and therefore lessen their use of value-free random exploration. Such a process seems to be imbalanced in youths with ADHD symptoms, as they show increased levels of value-free random exploration, which leads to suboptimal decision making.

## Supplementary Information

ESM 1 (DOCX 3.12 MB)
