# Supplementary material for: Exploration heuristics decrease during youth
Source: Cogn Affect Behav Neurosci. 2022 May 19;22(5):969–83. doi: 10.3758/s13415-022-01009-9 (PMC9458685; doi:10.3758/s13415-022-01009-9)
Supplement: Supplementary file 1 — (DOCX 3.12 MB) [file 13415_2022_1009_MOESM1_ESM.docx]

**Supplementary Material**

# Model descriptions

In this study, we use the models that were developed and validated for this task (Dubois et al., 2021). Here, we re-print these equations for completion. For a summary of the parameters of each model cf. Table S2. The value of each bandit is represented as a distribution $N(Q,S)$ with $S=0.8$. Subjects have prior beliefs about bandits’ values which we assume to be Gaussian with mean $Q_{0}$ (prior mean; free parameter) and uncertainty $\sigma_{0}$ (prior variance; free parameter).

**Mean and variance update rules**

At each time point 𝑡, in which a sample 𝑚, of one of the bandits is presented, the expected mean $Q$ and precision $\tau=\frac{1}{\sigma^{2}}$of each bandit are updated as follows:

$$Q_{i,t+1}=\frac{\tau_{i,t}* Q_{i,t}+\tau_{samp}*m}{\tau_{i,t}+ \tau_{samp}}$$

$$\tau_{t+1}^{i}=\tau_{samp}+ \tau_{t}^{i}$$

with $\tau_{samp}=\frac{1}{S^{2}}$the sampling precision, $S=0.8$ the fixed sampling variance, $m$ the presented sample, $i$ is the bandit and $t$ the time point. Those update rules are equivalent to using a Kalman filter (Bishop, 2006) in stationary bandits.

**Models**

We examined three base models reflecting complex exploration strategies: the UCB model, the Thompson model and the hybrid model. The UCB model encompasses the UCB algorithm (captures directed exploration) and a softmax choice function (captures the so-called value-based random exploration). The Thompson model reflects Thompson sampling (captures an uncertainty-driven value-based exploration). The hybrid model captures the contribution of the UCB model and the Thompson model, essentially a mixture of the above. We will compute three extensions of each model by either adding value-free random exploration ($c_{vf}$,$c_{n}$)={1,0}, novelty exploration ($c_{vf}$,$c_{n}$)={0,1} or both heuristics ($c_{vf}$,$c_{n}$)={1,1}. This leads to a total of 12 models (see the labels on the x-axis in Fig. 3). A coefficient $c_{vf}$=1 indicates that a ϵ-greedy component was added to the decision rule, ensuring that once in a while (every ϵ % of the time), another option than the predicted one is selected. A coefficient $c_{n}$=1 indicates that the novelty bonus $\eta$ is added to the computation of the value of novel bandits and the Kronecker delta $\delta$ in front of this bonus ensures that it is only applied to the novel bandit. For each model, the probability of choosing a bandit is described below. Please note that these three complex models make relatively similar predictions in our task, and that our model selection is primarily targeted at establishing the presence of exploration heuristics (in addition to complex exploration strategies).

UCB model:

In this model (Auer, 2003), an information bonus $\gamma$ is added to the expected mean $Q$ of each option, scaling with the option’s uncertainty $\sigma$.

$$P\left( i \right)=\frac{e^{\beta V_{i,t}}}{\sum_{x} e^{\beta V_{i,t}}}*\left( 1-c_{vf}\epsilon\right)+c_{vf}\frac{\epsilon}{3}$$

$$with V_{i,t} =Q_{i,t}+\gamma\sigma_{i,t}+c_{n}\eta\delta_{\left[ i=novel \right]},$$

$\gamma$ the information bonus, $V$the expected value, $\eta$the novelty bonus and $\beta$ the inverse temperature of the softmax (lower values producing more stochasticity).

Thompson sampling model:

In this model (Thompson, 1933), the overall uncertainty can be seen as a more refined version of a decision temperature (Gershman, 2018). At each time step, a sample $x_{i,t}\mathcal{\sim N(}V_{i,t}, \sigma_{i,t}^{2})$ is taken from each bandit. The probability of choosing a bandit i depends on the probability that all pairwise differences (contained in the two-dimensional vector $u$) between the sample from bandit $i$ and the other bandits $j\neq i$ were greater or equal to 0.

$$P\left( i \right)=P\left( \forall j:x_{i,t}>x_{j,t} \right)*\left( 1-c_{vf}\epsilon\right)+c_{vf}\frac{\epsilon}{3}$$

$$P\left( i \right)=\int_{0}^{\infty} \int_{0}^{\infty} ɸ\left( u;M_{i,t}, C_{i,t} \right) du*\left( 1-c_{vf}\epsilon\right)+c_{vf}\frac{\epsilon}{3}$$

$${{with M}_{i,t}=A}_{i}\left( \begin{matrix} V_{1,t} \\ V_{2,t} \\ V_{3,t} \end{matrix} \right), C_{i,t}=A_{i}\left( \begin{matrix} \sigma_{1,t} & 0 & 0 \\ 0 & \sigma_{2,t} & 0 \\ 0 & 0 & \sigma_{3,t} \end{matrix} \right)A_{i}^{T},$$

$$V_{i,t}=Q_{i,t}+c_{n}\eta\delta_{\left[ i=novel \right]},$$

$ɸ$ the multivariate Normal density function, $A$ the matrix computing the pairwise differences for each bandit and $x_{i,t}\mathcal{\sim N(}V_{i,t}, \sigma_{i,t}^{2})$ a sample taken from each bandit.

Hybrid model:

This model allows a combination of the UCB model and the Thompson model (Gershman, 2018).

$$P\left( i \right)=\left( wP_{UCB}\left( i \right)+\left( 1-w \right)P_{Thompson}\left( i \right) \right)*\left( 1-c_{vf}\epsilon\right)+c_{vf}\frac{\epsilon}{3}$$

with $w$the contribution of the UCB and Thompson sampling models

**Parameter estimation**

To fit the parameter values, we used the maximum a posteriori probability (MAP) estimate. The optimisation function used is fmincon in MATLAB. All the parameters besides $Q_{0}$ and $w$ were free to vary as a function of the horizon as they capture different exploration forms: directed exploration (information bonus $\gamma$; UCB model), novelty exploration (novelty bonus$\eta$), random exploration (inverse temperature $\beta$; UCB model), uncertainty-directed exploration (prior variance $\sigma_{0}$; Thompson model) and value-free random exploration ($\epsilon$-greedy parameter). The prior mean $Q_{0}$ was fitted to both horizons together as we did not expect the belief of how good a bandit is to depend on the horizon. The same holds for $w$, as we assume that the arbitration between the UCB model and the Thompson model does not depend on the horizon. The parameters were permitted to vary within the following bounds:$\sigma_{0}=\left[ {10}^{-8}, 8 \right], Q_{0}=\left[ 1, 10 \right], \epsilon=\left[ 0, 1 \right], \eta=\left[ 0, 5 \right], \gamma=\left[ 0, 0.5 \right], \beta=\left[ 1.4, 5 \right], w=[0, 1]$. The prior distribution used for the prior mean $Q_{0}$ and the prior variance$\sigma_{0}$ parameters were the normal distributions that approximate the generative distributions: $Q_{0} \sim N\left( 5, 2 \right)$ and $\sigma_{0} \sim N(1.4, 1)$. For the $\epsilon$-greedy parameter, the novelty bonus$\eta$ and the prior variance $\sigma_{0}$, a uniform distribution was used (equivalent to performing maximum likelihood estimation).

**Model comparison**

We performed a K-fold cross-validation with K=6. We partitioned the data of each subject into K folds (i.e. subsamples). For model fitting in our model selection, we used maximum likelihood estimation (MLE), where we maximised the likelihood for each subject individually (fmincon was ran with eight randomly chosen starting point to overcome potential local minima). We fitted the model using K-1 folds and validated the model on the remaining fold. We repeated this process K times, so that each of the K fold is used as a validation set once, and averaged the likelihood over held out trials. We did this for each model and each subject and averaged across subjects. The winning model was given by the exceedance probabilities using Bayesian Model Selection and was the Thompson sampling with ($c_{vf}$,$c_{n}$)={1,1}. (Fig. 3).

**Parameter recovery**

To make sure that the parameters are interpretable, we performed a parameter recovery analysis. For each parameter, we took four values, equally spread, within a reasonable parameter range (σ0=[0.5,2.5],Q0=[1,6],ϵ=[0,0.5],η=[0,5]). All parameters but Q0 were free to vary as a function of the horizon. We simulated behaviour with one artificial agent for each $4^{7}$ combinations using a new trial for each. The model was fitted using MAP estimation (cf. Parameter estimation) and analysed how well the generative parameters (generating parameters in Fig.5b) correlated with the recovered ones (fitted parameters in Fig. 5b) using Pearson correlation. Overall the parameters were well recoverable. Model parameters have also been found to be well recoverable in previous simulation analyses, whether using a similar parameter sampling method (Dubois et al., 2021), sampling from participants’ fitted parameters (Dubois & Hauser, 2021), or sampling from the mean and variance of participants’ fitted parameters (Dubois & Hauser, 2021).

|  | Children | Early adolescents | Late  adolescents |  |
| --- | --- | --- | --- | --- |
| Age | 9.32 (.27) | 13.13 (.30) | 17.18 (.30) |  |
| Gender (M/F) | 10/16 | 17/21 | 14/19 | F(2, 94)=.121, p=.886, $\eta^{2}$=.003 |
| Intellectual abilities | 93.88 (12.88) | 98.50 (13.86) | 97.45 (10.47) | F(2, 94)=1.095, p=.339, $\eta^{2}$= .023 |
| ADHD symptoms | 59.23 (13.41) | 56.05 (12.90) | 54.18 (11.30) | F(2, 94)=1.191, p=.308, $\eta^{2}$=.025 |

**Table S1.**

Characteristics of age groups. The age groups did not differ in gender, intellectual abilities (short form of the WASI-II including the Vocabulary and Matrix Reasoning subtest) nor ADHD symptoms (Conners 3AI-SR, Conners 2008). Mean (SD).

|  | **Model** | **Thompson** | | | | **UCB** | | | | **Hybrid** | | | |
| --- | --- | --- | --- | --- | --- | --- | --- | --- | --- | --- | --- | --- | --- |
|  |  |  | $+ \epsilon$ | $+ \eta$ | $+\epsilon+\eta$ |  | $+ \epsilon$ | $+ \eta$ | $+\epsilon+\eta$ |  | $+ \epsilon$ | $+ \eta$ | $+\epsilon+\eta$ |
| **Parameters** | Horizon independent | $Q_{0}$ | $Q_{0}$ | $Q_{0}$ | $Q_{0}$ | $Q_{0}$ | $Q_{0}$ | $Q_{0}$ | $Q_{0}$ | $w,Q_{0}$ | $w,Q_{0}$ | $w,Q_{0}$ | $w,Q_{0}$ |
|  | Horizon dependent | $\sigma_{0}$ | $\sigma_{0},\epsilon$ | $\sigma_{0},\eta$ | $\sigma_{0},\epsilon,$  $\eta$ | $\gamma,\beta$ | $\gamma,\beta,$  $\epsilon$ | $\gamma,\beta,$  $\eta$ | $\gamma,\beta,$  $\epsilon,\eta$ | $\sigma_{0},\gamma,$  $\beta$ | $\sigma_{0},\gamma,$  $\beta,\epsilon$ | $\sigma_{0},\gamma,$  $\beta,\eta$ | $\sigma_{0},\gamma,$  $\beta,$  $\epsilon,\eta$ |

**Table S2. Parameters of each model.** Table of parameters used for each model compared during model selection (cf. Fig. 3). Each of the 12 columns indicate a model. The three ‘main models’ studied were the Thompson model, the UCB model and a hybrid of both. Variants were then created by adding the $\epsilon$-greedy parameter, the novelty bonus and a combination of both. All the parameters besides $Q_{0}$ and w were fitted to each horizon separately. Parameters: $Q_{0}$=prior mean (initial estimate of a bandits mean); $\sigma_{0}$=prior variance (uncertainty about $Q_{0});$ $w$=contribution of UCB vs Thompson; $\gamma$ =information bonus; $\beta$=softmax inverse temperature; $\epsilon$=$\epsilon$-greedy parameter (stochasticity); $\eta$=novelty bonus.

| **Measure** | **Main effect** | | | **Interaction** |
| --- | --- | --- | --- | --- |
|  | **Horizon** | **Age** | **IQ** | **Age-by-horizon** |
| High-value bandit | F(1,94)=24.017,  p<.001, η²=.204 | F(1,94)=2.183,  $p_{\mathrm{cor}}$=.429, $p_{\mathrm{unc}}$=.143, η²=.023 | F(1,94)=2.945,  p=.089, η²=.03 | F(1,94)=2.462,  p=.12, η²=.026 |
| Low-value bandit | F(1,94)=8.62, p=.004, η²=.084 | F(1,94)=4.467,  $p_{\mathrm{cor}}$=.111, $p_{\mathrm{unc}}$=.037, η²=.045 | F(1,94)=6.655,  p=.011, η²=.066 | F(1,94)=0.019,  p=.89, η²<.001 |
| Novel bandit | F(1,94)=1.63, p=.205, η²=.017 | F(1,94)=0.014,  $p_{\mathrm{cor}}$=1, $p_{\mathrm{unc}}$=.905, η²<.001 | F(1,94)=0.238,  p=.627, η²=.003 | F(1,94)=2.227,  p=.139, η²=.023 |
| Prior variance $\sigma_{0}$ | F(1,94)=2.195, p=.142, η²=.023 | F(1,94)=0.123,  $p_{\mathrm{cor}}$=1, $p_{\mathrm{unc}}$=.727, η²=.001 | F(1,94)=2.683,  p=.105, η²=.028 | F(1,94)=0.09,  p=.765, η²=.001 |
| $\epsilon$-greedy parameter | F(1,94)=19.696, p<.001, η²=.173 | F(1,94)=5.583,  $p_{\mathrm{cor}}$=.06, $p_{\mathrm{unc}}$=.02, η²=.056 | F(1,94)=4.875,  p=.03, η²=.049 | F(1,94)=0.119,  p=.73, η²=.001 |
| Novelty bonus $\eta$ | F(1,94)=2.002, p=.16, η²=.021 | F(1,94)=0.406,  $p_{\mathrm{cor}}$=1, $p_{\mathrm{unc}}$=.526, η²=.004 | F(1,94)=0.045,  p=.832, η²<.001 | F(1,94)=3.372,  p=.069, η²=.035 |

**Table S3. Detailed statistics of task effects.** ANOVA results for behavioral and model parameter measures with the age group as between-subject factor (children, early adolescents, late adolescents), the decision horizon as within-subject factor horizon (long, short horizon) and IQ scores as covariate. ANOVA variables: F= F-statistic, p=p-value, η² = partial eta squared.

**Fig. S1. Visualisation of the 9 different sizes that the apples can take.** The associated rewards go from 2 (small apple on the left) to 10 (big apple on the right). The reward is given by linearly increasing the radius of the apple.


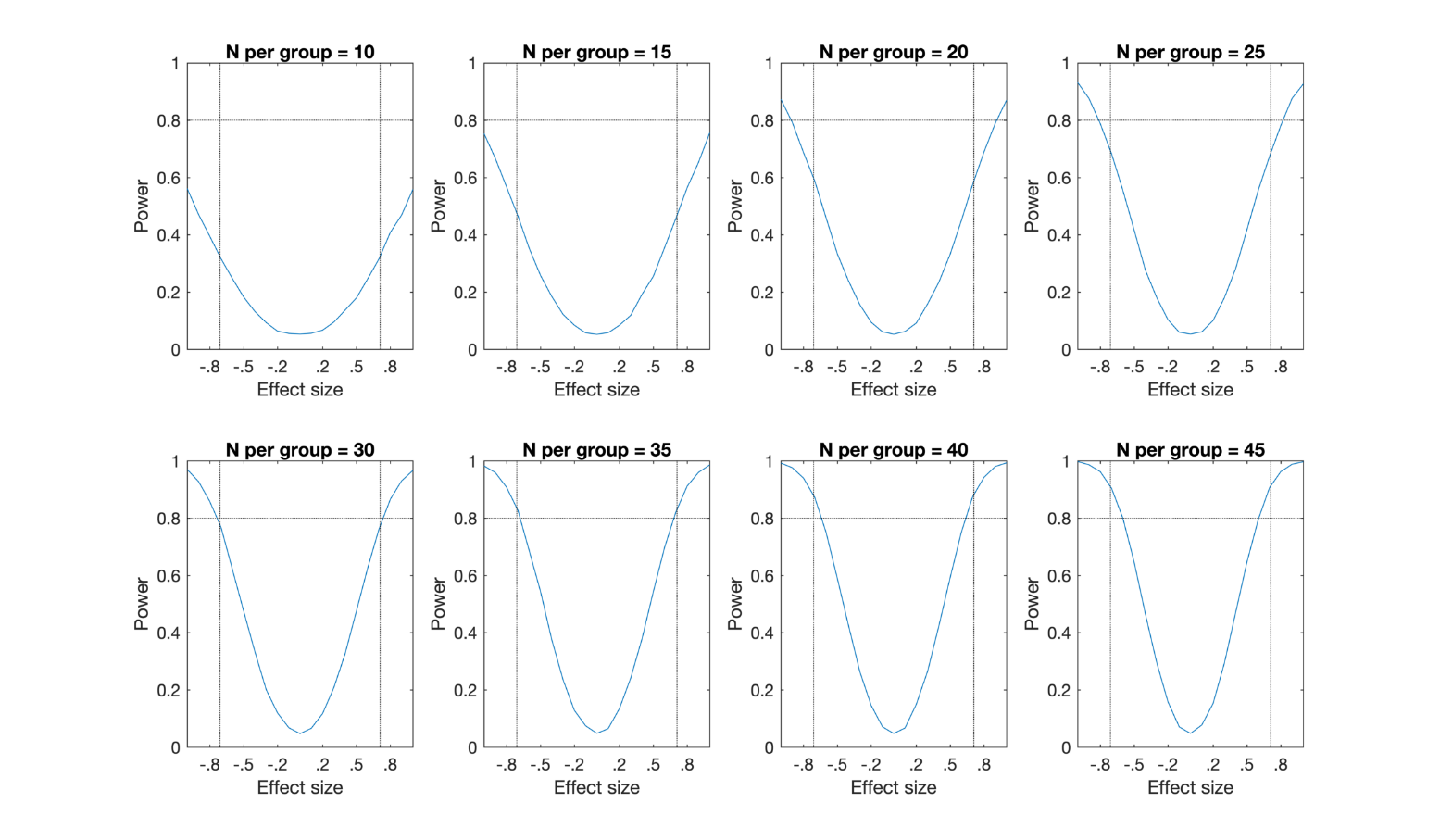
**Fig. S2. Power simulations.** To determine the sample size a power analysis was performed. Each subfigure represents the power curve for a different sample size. The power (y-axis) was computed for different effect sizes (x-axis). The horizontal dashed line indicates the desired power (=0.8). The vertical dashed lines (=.71) indicate the value obtained from the direct comparison between 2 independent groups of the $\epsilon$-greedy parameter in a similar study using the same task (Dubois et al., 2021). This value represents the lower-bound of the expected effect size as larger effect sizes are assumed in developmental samples. Those simulations indicate that a sample size of ~30 per group is enough to reach a statistical power of 0.8.


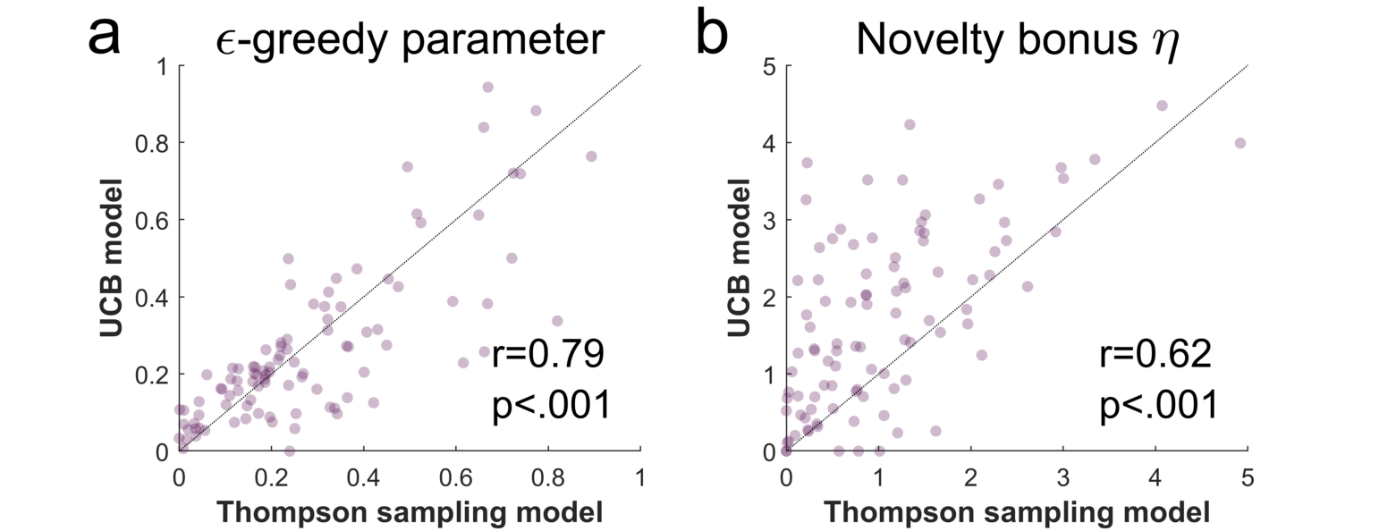


**Fig. S3. Parameter correlations across models.** Pearson correlation between parameter values from the Thompson+$\epsilon$+$\eta$ model and the UCB+$\epsilon$+$\eta$ model. (a) $\epsilon$-greedy parameter values. (b) Novelty bonus $\eta$ parameter values.


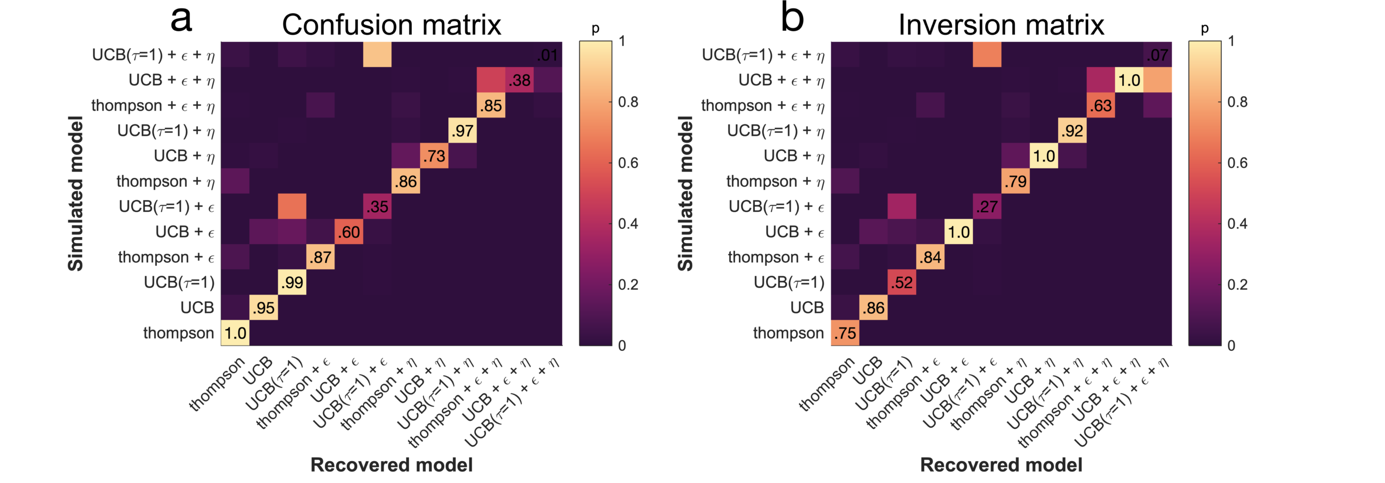


**Fig. S4. Model recovery.** Figure is re-printed for completion from a previous registered report (Dubois & Hauser, 2021). (a) Confusion matrix of model identification: p(fit model|simulated model). The percentage p of how often each fitted model won given the simulated model was computed (b) Inversion matrix of model identification: p(simulated model|fit model). The percentage p of how often the data was simulated by a specific model given the fitted model was computed.


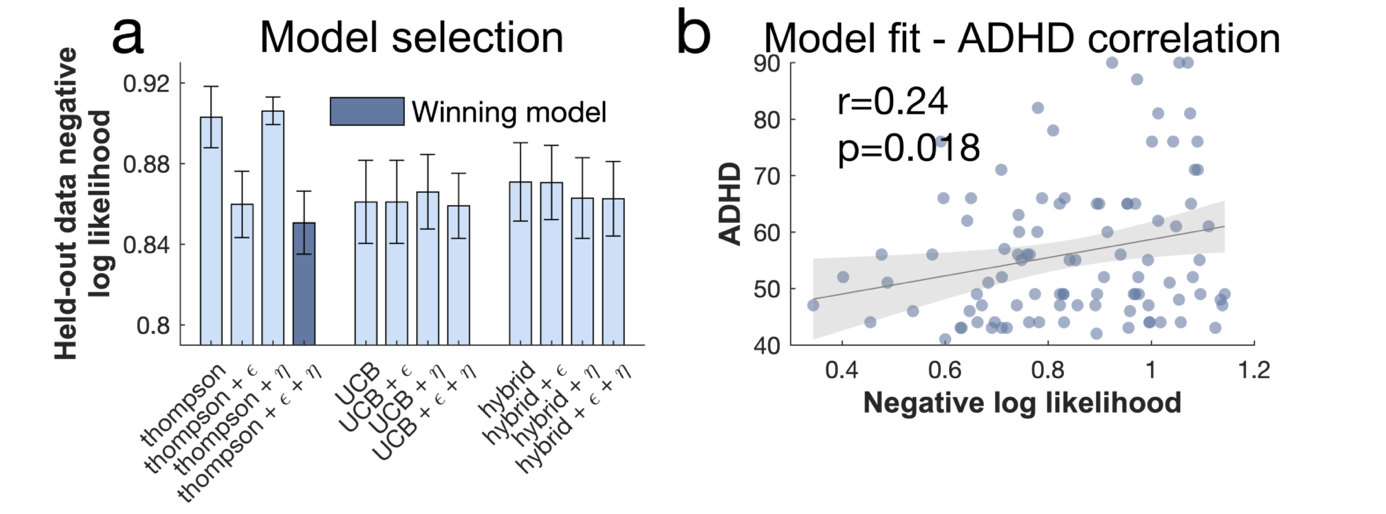


**Fig. S5. Supplementary model fit plots.** (a) Winning model in terms of negative log likelihood. (b) In line with previous studies (Moutoussis et al., 2021), we observed a correlation between the model fit (negative log likelihood measure) and our questionnaire measure (ADHD).


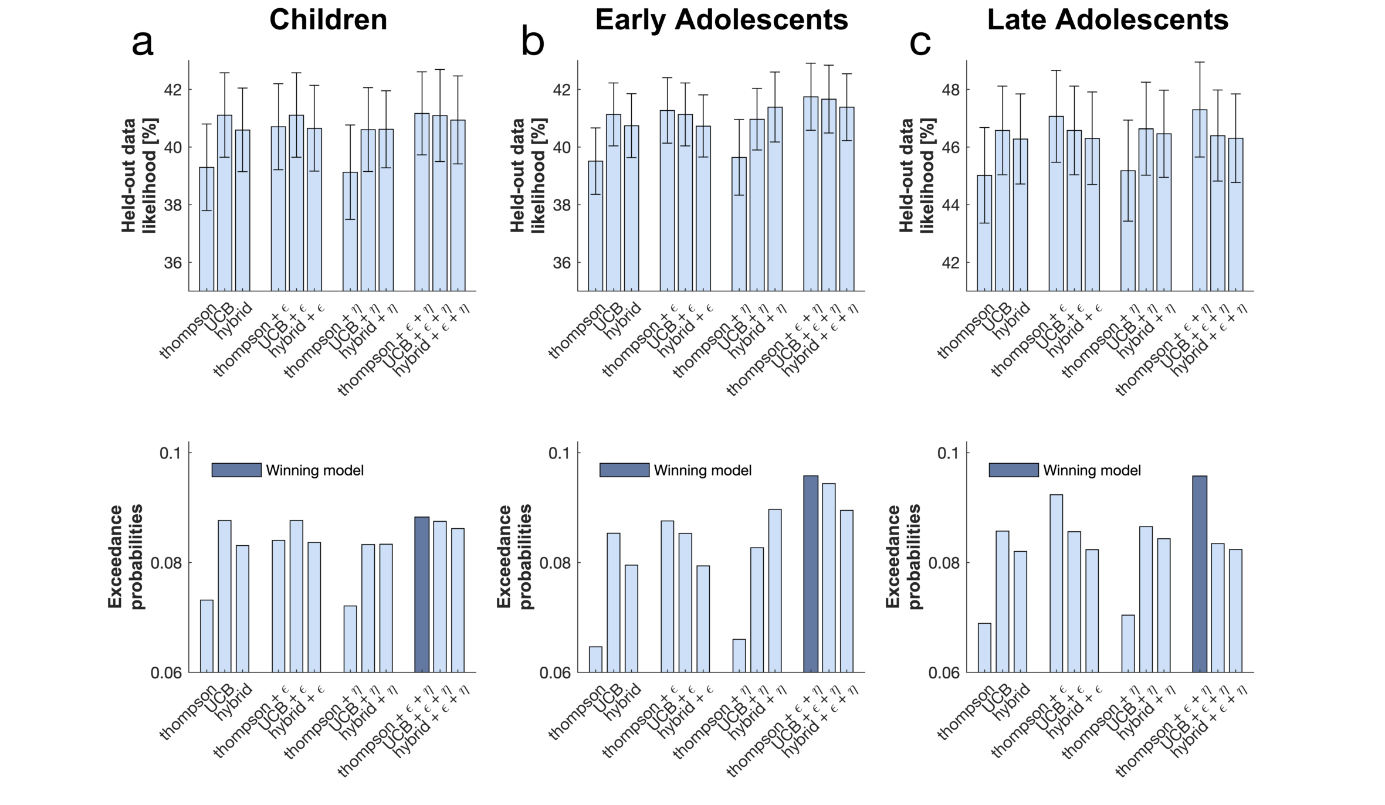


**Fig. S6. Model fit per age group.** The winning model (Thompson+$\epsilon$+$\eta$) was the same across all age groups: (a) children, (b) early adolescents, (c) late adolescents.
